# Supplementary material for: The interactome of intact mitochondria by cross-linking mass spectrometry provides evidence for coexisting respiratory supercomplexes
Source: Mol Cell Proteomics. 2017 Dec 8;17(2):216–32. doi: 10.1074/mcp.RA117.000470 (PMC5795388; doi:10.1074/mcp.RA117.000470)
Supplement: Supplemental Data [file supp_RA117.000470_133922_0_supp_23978_fzffwf.zip › spectra_annotation/mito_spectra_annotation/154-1-11-1-15-1.pdf]

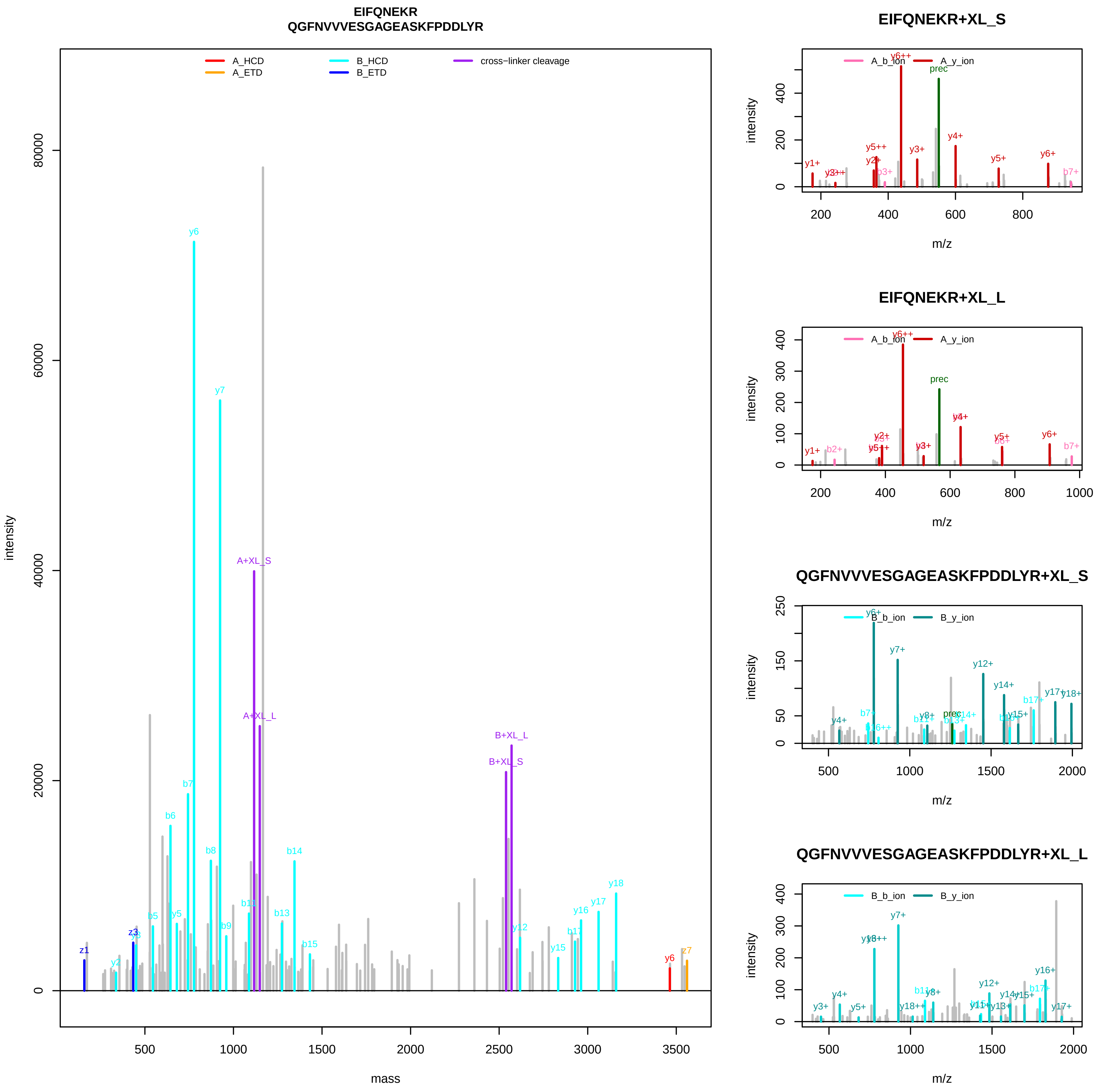

EIFQNEKR
QGFNVVVESGAGEASKFPDDLYR
A_HCD
A_ETD
B_HCD
B_ETD
cross−linker cleavage
intensity
mass
0
20000
40000
60000
80000
500
1000
1500
2000
2500
3000
3500
z1
z3
y2
b5
b6
y5
b7
y6
y7
b8
b9
b11
A+XL_S
A+XL_L
b13
b14
b15
B+XL_S
B+XL_L
y12
y15
b17
y16
y17
y18
y6
z7
EIFQNEKR+XL_S
A_b_ion
A_y_ion
prec
m/z
y1+
y2+
y3+
y4+
y5+
y6+
y5++
y6++
b3+
b7+
EIFQNEKR+XL_L
b2+
QGFNVVVESGAGEASKFPDDLYR+XL_S
B_b_ion
B_y_ion
y4+
y6+
y7+
y8+
y12+
y14+
y15+
y17+
y18+
b7+
b11+
b13+
b14+
b17+
QGFNVVVESGAGEASKFPDDLYR+XL_L
y3+
y13+
y16+
